# Nitric Acid and Caries

**Published:** 1859-01

**Authors:** 


### EDITORIAL DEPARTMENT.

*Nitric Acid and Caries.*—The Dental Register of the West is laboring very hard to force a controversy upon us, because we exposed some absurd chemical errors of one of its editors. Now we wish it to be understood once for all, that we shall, at all times, comment as we see proper, upon whatever opinions may be advanced in reference to dental science, and this, too, without considering ourselves at all obliged to enter into a defence of our criticisms in case the writer commented upon chooses to assail our opinions. A contrary course would involve us in endless and unprofitable controversy.

As a general rule, we are disposed to avoid controversy in our pages. There are several reasons for this. In the first place, the readers of one journal are not always readers of another, and consequently garbled and partial statements of the arguments advanced may be put forward by either side, without the chance of correction. Again, we do not ourselves consider it a fascinating employment to read perpetually one side of a discussion, without seeing any more of the other than those portions which the disputant thinks easy of refutation; and we infer that our readers may be similarly disposed.

A still graver objection is, that we do not choose to put ourselves upon the level of every scribbler who may desire to cross swords with us. It is the height of folly to attempt a discussion with a man, who, being ignorant of the science involved in the question, fails at once to see the point of the arguments addressed to him, or to advance any which are worthy the trouble of a refutation. The disadvantage of this sort of discussion is well set forth in Dr. Holmes' "Autocrat of the Breakfast Table."

"If a fellow," says that witty writer, "attacked my opinions in print, would I reply? Not I. Do you think I don't understand what my friend, the Professor, long ago called *the hydrostatic paradox of controversy?* Don't know what that means? Well, I will tell you. You know, that, if you had a bent tube, one arm of which was of the size of a pipe stem, and the other big enough to hold the ocean, water would stand at the same height in the one as in the other. Controversy equalizes fools and wise men in the same way—*and the fools know it.*"

So much for general principles. In the particular case before us, we have additional reasons for refusing a controversy. The first article in the Register could not be noticed by us without a forfeiture of self-respect. We disdained to reply to the scurrilous personalities by which the editor of that journal labored to divert public attention from his own chemical short-comings. To one passage alone of that delectable production we will refer in passing, that in which he charges us with *jealousy.* We beg to set his mind entirely at rest in regard to that matter, by assuring him that we had lost all consciousness of his existence, till his chemical quackery reminded us of a not very honorable notoriety which he obtained a few years since in connection with A. J. Watts' sponge gold. (See Dr. Dwinelle's article at the close of the number of this Journal for April, 1855.)

We depart, in this one instance, from our usual custom, because we wish to be a little more explicit upon the subject of the probability of nitric acid being formed in the mouth. That our readers may not suspect us of wishing to present them but one side of the question, we copy the entire article of the Register.

"*The American Journal* is again in trouble on the chemistry of caries. It says, in reference to the discussions on this subject at Cincinnati, 'the unfounded theories about nitric acid in the mouth played a conspicuous part;' and again, 'it was said that nitric acid produced upon teeth out of the body, appearances resembling white decay.'

No doubt the above was said, for the American Journal says so, but we are utterly at a loss to know who said it. It was probably said only in a whisper; for we were present at all the discussions, and listened with a good degree of interest. Indeed, about the only member of the convention that said anything on the subject was *ourself*, known as the "anonymous friend" of the Journal. And if the above quotation has any allusion to our remarks, we have only to say that we did not speak of the action of nitric acid *out of the body*; for our experiments were tried *in the mouth* as well as out of it. And we did not then, and do not now claim "that nitric acid is the sole agent in the production of the white decay;" but we did claim that nitric acid is capable of producing white decay either in or out of the mouth, and we stated then that as yet we "knew of nothing else capable of producing it." Will the American Journal have the kindness to tell us of something else capable of producing it?

The Journal tells us "there are, indeed, very good reasons for doubting the presence of free nitric acid in the mouth. The hypothesis of the conversion of ammonia into nitric acid will not answer at all. The conditions under which this change takes place do not exist in the mouth."

Well, doctors differ. It maintains on the authority of Bischoff, that "nitric acid in a free state, is formed in nature, only when an electric spark passes through oxygen and nitrogen."

Liebig tells us that the last products of the decay and putrefaction of animal bodies are *ammonia*, in the temperate and cold climates, and *nitric acid* in the tropics and hot climates, and he adds that the latter is the product of the transformation of the former. The mouth is not a *tropic*, but the Journal will agree that its *climate* is warm.

Liebig also tells us that, "when moist azotized animal matter is exposed to the action of the air, ammonia is liberated;" and again he says "ammonia cannot be exposed to the action of oxygen without the formation of an oxyd of nitrogen, and in consequence the production of nitric acid." This latter idea is variously stated by him, as for exampled, "nitric acid is always a product when ammonia is present in the substance exposed to oxydation."

From this it is evident that Liebig believes that the hypothesis of the conversion of ammonia into nitric acid *will answer*, notwithstanding the contrary opinion of the Journal.

In speaking of this transformation of ammonia, the Journal says, "It is no easy matter, to accomplish this result;" but Liebig says, "It is owing to the *great facility* with which ammmonia is converted into nitric acid, that it is so difficult to obtain a correct determination of the quantity of nitrogen in a compound subjected to analysis, in which it is either contained in the form of ammonia, or from which ammonia is formed by an elevation of temperature."

The Journal says of this transformation that "the surrounding deoxydizing influences render it almost impossible that it could occur" in the mouth.

The *deoxydizing* powers of the mouth may sound strange to those dentists who use oxydizable metals either for plates or fillings; but then they must yield the point that these powers are quite formidable, for the Journal is not likely mistaken, though it did tell us that none but cane sugar is used in food. But in what do these deoxydizing influences consist? Are they explained by the fact that the saliva absorbs oxygen readily from the air and *imparts it to other bodies?* that, on this principle, it oxydates gold and silver, when triturated with it? (See Piggot's Chemistry p. 172 ) Now, every dental student knows that substances are oxydized more readily in the mouth than in the atmosphere; and we conclude, therefore, that the junior editor must have indited this, for the senior, being a practical dentist, would know better than to make such a statement.

It is pretty evident the Journal and we differ in regard to the chemistry of caries. We have only to say that on our part, and we presume on theirs too, there is an honest difference. And though we have not referred to many authorities in these remarks, it is not that they are scarce, but that brevity is desirable. The positions of Liebig above noticed are sustained by chemists generally—not by all we are well aware. And if these positions are true, we contend that we have given the most rational account of "white decay" on record. Every dentist knows that decay is usually a gradual process; and every chemist knows that the constant formation of nitric acid for a length of time, in quantities too small to be recognized by chemical tests, would be sufficient to produce the disorganization of the tooth. And if the same state of circumstances on a large scale invariably produces nitric acid, we have a right to infer that these circumstances, on a reduced scale, will produce the same results, correspondingly reduced.

In conclusion we do hope the Journal will tell us what does produce dental decay in its various forms, or at least attempt to give us some rational account of the matter. One of the editors has told us that "the different acids generated during the putrefaction or fermentation of vegetable or animal food are fully capable of disorganizing the teeth." Is this true of carbonic acid? one of the acids generated. (See Piggot's Chemistry, p. 157.) Now we submit that it is time we had something of an affirmative nature from the Journal. It has been negative long enough. It is easy for it to deny the soundness of our positions. Will it give us its own? W.

It will be seen that this is, in no sense, a reply to the positions we took in our remarks upon the nitric acid hypothesis of caries.

Our objections were that nitric acid, so far as known, is formed in combination with a base, and in consequence of the chemical tendencies communicated by the presence of that base; that consequently, if nitric acid were formed in the mouth from decaying substances, it would not be *free*, and therefore incapable of acting upon the teeth in the manner assumed; that the vitrification of ammonia could not take place in the mouth, and we might have added that if it did, the nitric acid would occur as it does in rain-water in the form of nitrate of ammonia and not of free nitric acid. Now these being positives, which will be conceded by people who understand chemistry, there is seen to be a strong antecedent improbability that nitric acid, in a free state, can be formed in the mouth. Against this there can be but one successful argument, the actual demonstration of the acid. Prove its presence, and we have not another word to say. But we still insist that this has not been demonstrated, and in the last paragraph but one of W's article, we have the extorted confession that it cannot be done. The whole argument therefore, is one of bare probabilities, which may be summed up in the following elegant, conjectural sorites: "*If* ammonia be generated in the mouth in a free state, perhaps it may be converted into nitric acid; *if* nitric acid is formed, it may be free; if free, it may not find alkalies to neutralize it, and may act upon the teeth, *therefore*, nitric acid is a cause of white decay." "Oh, most lame and impotent conclusion!" If our readers do not like the argument, they must quarrel with the editor of the Dental Register of the West, for it is but an abridged form of his elaborate attempt at logic.

The editor has been so busy in attempting to correct our chemistry, that he has forgotten to attend to his own. He should procure a new edition of Liebig, and not quote from the old lucubrations of a dozen years ago. Chemistry is a rapidly advancing science, and requires no little assiduity on the part of those who would keep up with it.

Let us look for a moment at the present state of the nitrification question. We shall not quote the old and well known observations of Kuhlman, made in 1839, though W. might possibly learn something from them, for example, that if ammonia forms nitric acid when mixed with atmospheric air in the presence of alkalies, on the other hand, nitric acid in the presence of hydrogen in the hydracids, is converted into ammonia Nor shall we quote Bischoff, with whose authority among chemists we did not expect the "anonymous friend" of the Journal to be acquainted.

The celebrated English agricultural chemist, Mr. Way, in his admirable paper on drainage water, tells us that he is very unwilling to believe in the conversion of ammonia into nitric acid in the soil. "It is by no means easy," says he, "to produce this result when the ammonia is out of the sphere of other influences, and the strongest oxydating substances are unable to effect it."

M. Cloez (*Comptes Rendus*, Nov. 26th, 1855) shows that atmospheric nitrogen, free from ammonia, may, under certain circumstances, with the aid of porous bodies, give rise to the formation of nitrates.

De Luca, following out this idea, has shown that humid ozonized air, free from ammonia, gives rise, in the presence of alkalies, to nitrates.

These experiments indicate the source of the error in early observations, which attribute this appearance of nitrates to the oxydation of ammonia.

One of the most recent studies of nitrification has been made by Desmarest, who (*Comptes Rendus*, Aug. 11, 1856,) gives, in the synopsis of his conclusions, the following opinion in reference to this matter.

"That nitric acid is not formed by the oxydation of the nitrogen of ammoniacal gas or of organic matter, at the expense of the oxygen of the air. That, finally, it is formed only when nitrogen is found in the presence of an excess of oxygen, that is to say, in a case which does not ordinarily occur in nature."

It will be seen by these various statements that the question is in a very unsettled state, and that the whole tendency of modern research is in the direction intimated in our last article upon this subject.

The editor fails to comprehend the deoxydating agencies to which we alluded. This does not surprise us. Had he been a chemist he would have understood us. He would have known that any substance, undergoing oxydation, robs of oxygen all those with which it is in contact, and that this is a peculiarity of which the analytical chemist daily avails himself. Now the process of putrefaction is one of gradual oxydation, commencing with the formation of a number of substances which eagerly imbibe oxygen, and therefore *deoxydate.* Of course we were not talking about saliva, and if the editor really thinks we were, he exhibits a fund of ignorance greater than we had given him credit for. The whole question was about the process of putrefaction, in which the new chemical light rising in the West, showed us first ammonia, then free nitric acid, resulting from its oxydation and dissolving the phosphate of lime of the teeth to cause white caries.

A discussion in reference to nitrification, not of ammonia, but of nitrogenous animal matters, took place last year in the French Academy. Pelouze remarked that the nitrates are *destroyed* under the influence of *putrescent animal matter*, since saltpetre mixed with white of egg slowly disappears, the acid of the salt being changed into ammonia. Boussingault, one of whose papers gave rise to the discussion, avowed himself of the same opinion as Pelouze, and acknowledged that when there is a preponderance of nitrogenous matter, nitrification ceases, and then names precisely the conditions which we mentioned in our last article, as indispensable to the formation of nitrates. Perhaps these chemists may be as little known to W. as was Bischoff. If so, we have only to refer him to some intelligent apothecary's boy.

We must resist the appeal of W for information as to the action of acids. How conscious soever we may be of his need of such knowledge, we cannot afford to devote our pages to his instruction. We must refer him to elementary works on the science.

W. makes a great ado over our having said that the saccharine substances used in food are all made of cane sugar. Any candid person would see at once that this was a slip of a too hurried pen, but W. has so little to plume himself upon that it would be a pity to deprive him of this great theme of rejoicing.

It is not likely that we will soon again step out of our way to notice W. He attracted our attention in the first instance by meddling with things above his comprehension. We should not then have noticed him publicly, were it not that his crudities were paraded as part of the proceedings of one of our societies, that we suspected they would be reproduced in Europe, and that we anticipated a storm of ridicule which would fall not upon him, but upon the whole dental profession in America. To avert this by showing the world that they were not received here, was the sole object of our first article. *Ne sutor ultra crepidam* is a sensible old adage which would save much trouble if generally acted on, and which we commend to W's serious consideration *

* Page 144, fourth line from bottom, read *nitrification* for *vitrification* : on last line, for *positives* read *positions.*

